# Relegating Species Descriptions to Electronic Supplementary Information Puts Critical Data for Taxonomy at Risk

**DOI:** 10.1002/ece3.72234

**Published:** 2025-09-28

**Authors:** Diana O. Fisher, Stephen M. Jackson, Kenny J. Travouillon, Linette S. Umbrello, Andrew M. Baker, Mark D. B. Eldridge, Greta J. Frankham, Tyrone H. Lavery, Australasian Mammal Taxonomy Consortium Steering Committee

**Affiliations:** ^1^ University of Queensland Brisbane Australia; ^2^ Australian Museum Sydney Australia; ^3^ Western Australian Museum Perth Australia; ^4^ Queensland University of Technology Brisbane Australia; ^5^ Queensland Museum Brisbane Australia; ^6^ University of Melbourne Melbourne Australia

## Abstract

There is a current trend to relegate the details of molecular and morphometric analyses in species descriptions to electronic Supplementary Information (eSI), where they may be separated and lost. We find that half of the species descriptions in non‐specialist journals since 2012 have put important material in eSI. The identity of specimens and measurements that are used in taxonomic descriptions needs to be available in perpetuity. A simple and effective way to avoid relegating species description details to electronic SI in non‐specialist journals is to publish these in appendices attached to the main text, rather than in separate digital files.

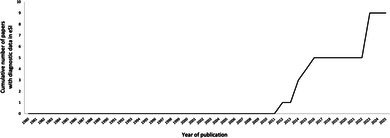

There is a current trend to relegate the details of molecular and morphometric analyses in species descriptions to electronic Supplementary Information (eSI). Reliable species description practice is the basis of organismal biology, conservation practice, and law. Material in species descriptions must therefore be available for scientific interrogation. This means not only collecting and safeguarding physical type specimens in museums (Gutiérrez and Pine 2017) but also making the identity of specimens and measurements that are used in taxonomic descriptions available in perpetuity. Measurements must be transparent and repeatable, so future researchers can check diagnoses and use all appropriate data to revise taxonomy (Gutiérrez and Pine 2017).

Placing important data only in electronic databases that are separate from the main text threatens this repeatability because electronic documents, including Digital Object Identifier links, are disappearing at an alarming rate (Brainard 2020; Wild 2024; Laakso et al. 2021). For example, more than a quarter of DOIs are not registered in a major archive (Eve 2024).

To assess the extent of the risks of placing species description data in a separate electronic SI, we analysed all of the 145 Australasian mammal species descriptions published since 1980 (before the electronic SI began) (AMTC 2024). Sixty‐two percent of these taxonomic descriptions are in specialist museum, systematics, or taxonomy journals, which publish around one species description per year on average. These specialist journals continue to place all important data for species diagnosis in the main text, figures, and tables of every description (Figure 1).

**FIGURE 1:**
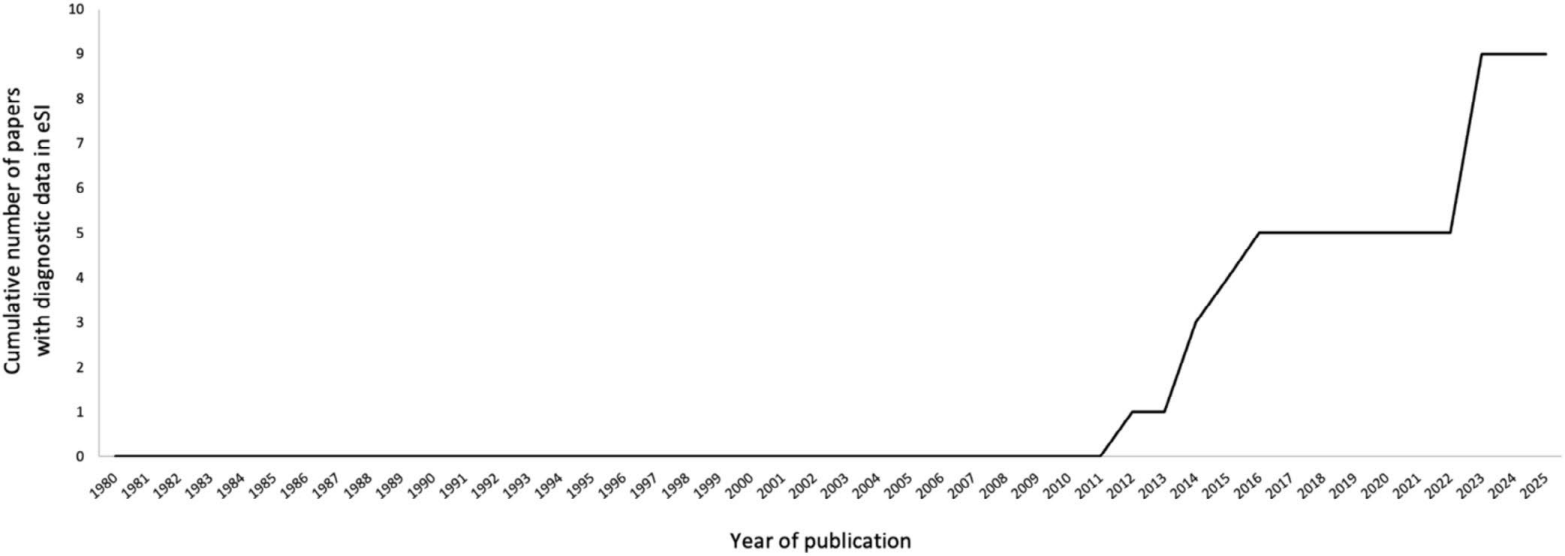
The number of Australasian mammal species descriptions published with diagnostic data only in electronic Supplementary Information (eSI) published per year in 1980–2025. All species descriptions published with diagnostic data only in eSI are in non‐specialist journals (not museum, systematics, and taxonomy journals).

The trend to relegate species description details to electronic SI began in 2012 (Figure 1) and is associated with publishing mammal species descriptions in non‐specialist journals. We find that since 2012, 50% of species description papers (9/18) published in non‐specialist journals (i.e., mammalogy, zoology, ecology, evolution, or biology journals) put some or all of the specimen identities and molecular and morphometric analyses in electronic SI. In 2023–2024, components of species descriptions were published only in eSI in half of the species descriptions across all journals.

The number and proportion of species descriptions published in journals with higher readership and thus higher impact factors have increased since the mid‐1990s (Figure 2). From 1980 to 1994, 20% of Australasian mammal species description papers were in non‐specialist journals; from 1995 to 2009, the percentage was 56%, and since 2010, 50% are in non‐specialist journals. The modal Journal Impact Factor (JIF) of specialist museum, systematics, and taxonomy journals that have published species descriptions since 2012 is 0.9, and in zoology, ecology, and evolution journals, the modal JIF is 2.3.

**FIGURE 2:**
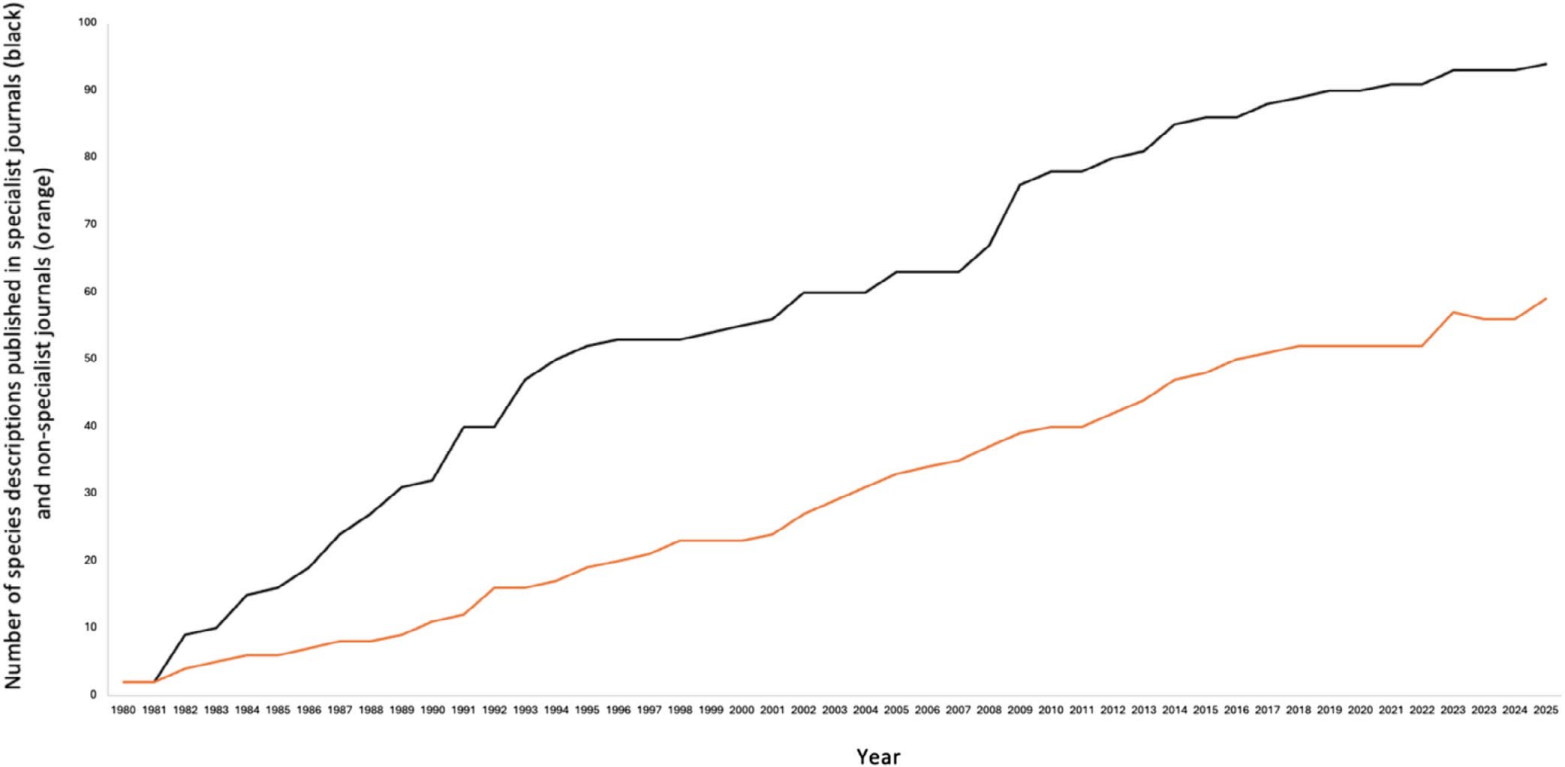
The number of Australasian mammal species descriptions published in specialist journals (black) and non‐specialist journals (orange) per year in 1980–2025.

A simple and effective way to avoid relegating species description details to electronic SI in non‐specialist journals is to publish these in appendices attached to the main text, rather than in separate digital files. To guard against loss of critical specimen information and measurements, we urge scientists to request that all critical data for taxonomic diagnosis be included in the main text of species descriptions or in an appendix published in the same document as the main text and downloaded in the same PDF file. Editors and journal policy can facilitate this. Some non‐specialist journals already have an editorial policy against electronic SI. For example, *Ecology and Evolution* author guidelines state, ‘*we discourage the use of supplementary material. It's traditional purpose is to save space … supplementary material housed separately from the paper are often lost (at worst) and rarely accessed (at best)*’.

## Author Contributions

**Diana O. Fisher:** conceptualization (equal), formal analysis (equal), investigation (equal), writing – original draft (equal), writing – review and editing (equal). **Stephen M. Jackson:** conceptualization (equal), formal analysis (equal), investigation (equal), methodology (equal), writing – original draft (equal), writing – review and editing (equal). **Kenny J. Travouillon:** conceptualization (equal), formal analysis (equal), investigation (equal), writing – original draft (equal), writing – review and editing (equal). **Linette S. Umbrello:** conceptualization (equal), formal analysis (equal), investigation (equal), writing – original draft (equal), writing – review and editing (equal). **Andrew M. Baker:** conceptualization (equal), formal analysis (equal), investigation (equal), writing – original draft (equal), writing – review and editing (equal). **Mark D. B. Eldridge:** conceptualization (equal), formal analysis (equal), methodology (equal), writing – original draft (equal), writing – review and editing (equal). **Greta J. Frankham:** conceptualization (equal), formal analysis (equal), investigation (equal), writing – original draft (equal), writing – review and editing (equal). **Tyrone H. Lavery:** conceptualization (equal), formal analysis (equal), investigation (equal), writing – original draft (equal), writing – review and editing (equal).

## Conflicts of Interest

The authors declare no conflicts of interest.

## Data Availability

Data derived from the following public domain resource: AMTC Australian Mammal Species List. Version 4.1. https://australianmammals.org.au/publications/amtc‐species‐list.

## References

[ece372234-bib-0001] AMTC . 2024. “AMTC Australian Mammal Species List. Version 4.1.” (2024–2025). https://australianmammals.org.au/publications/amtc‐species‐list.

[ece372234-bib-0002] Brainard, J. 2020. “Dozens of Scientific Journals Have Vanished From the Internet, and No One Preserved Them.” Science 369, no. 6509: 1278. 10.1126/science.abe6998.32913078

[ece372234-bib-0003] Eve, M. P. 2024. “Digital Scholarly Journals Are Poorly Preserved: A Study of 7 Million Articles.” Journal of Librarianship and Scholarly Communication 12: eP16288.

[ece372234-bib-0004] Gutiérrez, E. E. , and R. H. Pine . 2017. “Specimen Collection Crucial to Taxonomy.” Science 355, no. 6331: 1275. 28336633 10.1126/science.aan0926

[ece372234-bib-0005] Laakso, M. , L. Matthias , and N. Jahn . 2021. “Open is Not Forever: A Study of Vanished Open Access.” Journal of the Association for Information Science and Technology 79: 1099–1112.

[ece372234-bib-0006] Wild, S. 2024. “Millions of Research Papers at Risk of Disappearing From the Internet.” Nature 627: 256. 38438607 10.1038/d41586-024-00616-5

